# Syndrome de West: à propos d'une observation

**DOI:** 10.11604/pamj.2015.21.258.7414

**Published:** 2015-08-07

**Authors:** Marcellin Bugeme, Pascal Nawej, Olivier Mukuku

**Affiliations:** 1 Faculté de Médecine, Université de Lubumbashi, République Démocratique du Congo; 2 Centre Neuropsychiatrique Dr Joseph Guislain/Frères de la Charité, Lubumbashi, République Démocratique du Congo; 3 Centre médical du Centre-Ville de Lubumbashi (C.M.D.C.), Lubumbashi, République Démocratique du Congo

**Keywords:** Syndrome de West, spasmes infantiles, hypsarythmie, Lubumbashi, West syndrome, infantile spasms, hypsarrhythmia, Lubumbashi

## Abstract

Nous rapportons une observation d'un nourrisson de 7 mois présentant des spasmes infantiles et une microcéphalie. Elle était née à terme, en position de siège et avait présenté une asphyxie intrapartum (dépression néonatale) ayant conduit à une encéphalopathie néonatale modérée. Le diagnostic de Syndrome de West était confirmé à l’électroencéphalographie qui avait montré un tracé hypsarythmique. L'enfant avait été mise sous un traitement fait de Vigabatrine et son évolution était marquée par une régression des crises non seulement par rapport à l'intensité, mais aussi par rapport à la fréquence et à la durée. Sur le plan psychomoteur, nous avons noté un retard de développement.

## Introduction

Le syndrome de West fut décrit la première fois en 1841 par un chirurgien anglais William James West. Ses premières observations décrivaient les spasmes chez son propre fils, qui était âgé d'environ 4 mois à cette époque et il les avait nommés « tics de Salaam » [1]. L'incidence est de 2 à 3,5/10000 naissances vivantes, avec une apparition au cours de la première année de vie de l'ordre de 90% chez les personnes touchées. L’âge maximal d'apparition se situe entre 3 et 7 mois; l'apparition après 18 mois étant considérée rare [2].

## Patient et observation

**Figure 1:**
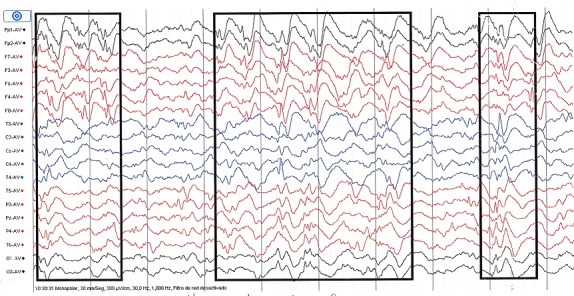
Image électroéncéphalographique montrant un tracé hypsarythmique

**Figure 2:**
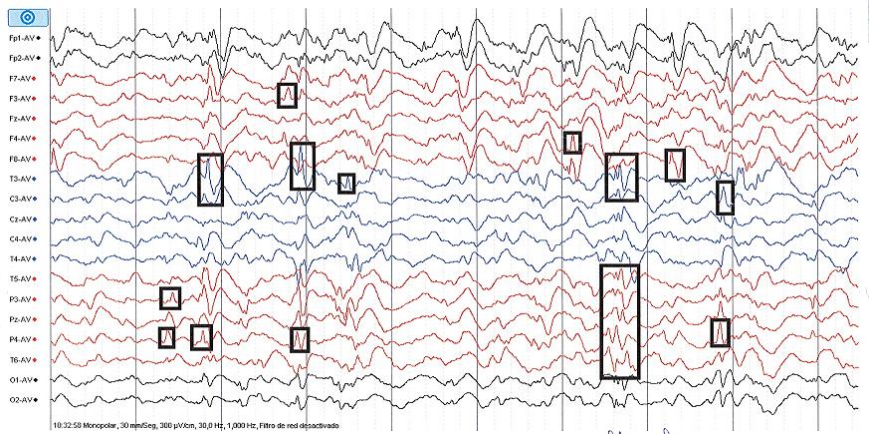
Image électroéncéphalographique montrant des pointes multifocales

Nourrisson Y.L, de sexe féminin et âgée de 7 mois, est amenée en consultation au Centre Médical du Centre Ville (CMDC) en date du 23 août 2014 pour crises convulsives. Ces crises avaient débuté à 4 mois de vie avant la consultation et n'avaient jamais été prises en charge. Dans ses antécédents périnataux, la patiente était née à terme, en position de siège et avait présenté une asphyxie intrapartum (dépression néonatale) ayant conduit à une encéphalopathie néonatale modérée. Les crises étaient décrites comme des contractures musculaires intéressant presque tout le corps, mais avec une prédominance aux membres inférieurs qui se plient en pleine crise. Un hochement de la tête avec une flexion de celle-ci sur la poitrine était souvent constaté. Les crises duraient environs 10 secondes avec une fréquence de plusieurs crises par mois. Sur le plan neurosomatique, on avait objectivé une microcéphalie (périmètre crânien = 40 cm) et une hypotonie axiale. Le reste de l'examen était sans particularité. L’électroencéphalogramme réalisé avait mis en évidence des pointes multifocales, asymétriques avec des zones de haut voltage et de bas voltage réalisant une hypsarythmie (Figure 1, Figure 2). Nous avions conclu à des spasmes infantiles ou Syndrome de West. L'enfant avait été mise sous un traitement fait de Vigabatrine (Sabril^®^) à la dose de 40 mg/kg/j. Son évolution était marquée par une régression des crises non seulement par rapport à l'intensité, mais aussi par rapport à la fréquence et à la durée. Sur le plan psychomoteur, nous avons noté un retard de développement.

## Discussion

Le syndrome de West est constitué d'une triade diagnostique caractéristique: survenue de salves de spasmes en flexion ou parfois en extension, régression du développement mental et observation en électroencéphalographie d'un tracé hypsarythmique [3]. Notre patiente présente une clinique typique de ce syndrome. L'hypsarythmie représente le critère diagnostique neurophysiologique primaire du syndrome. Selon Guzzetta, elle est associée à des perturbations des fonctions neurosensorielles et neurodéveloppementales, surtout lors du sommeil lent profond; état au cours duquel il a lieu la consolidation des acquis chez les enfants [4]. S'agissant de l’étiologie, chez notre patiente, la dépression néonatale était retenue comme cause. Dans notre milieu, la dépression néonatale sévère a été significativement associée à l'accouchement du siège par voie basse [5]. Dans l’étude de Hwang, l'encéphalopathie hypoxo-ischémique est une cause primaire dans 50,8% des cas [6]. Une récente étude menée en Inde trouve l'ischémie cérébrale périnatale comme cause dans 55% des cas [7]. Son diagnostic est établi en période périnatale et néonatale en s'appuyant sur des critères cliniques (dystocie lors de l'accouchement, des signes de souffrance foetale). Le devenir du syndrome de West est difficilement prévisible, cette encéphalopathie étant un grave syndrome souvent réfractaire au traitement et fréquemment associé à un retard mental [8]. Chez notre patiente, nous avons noté un retard de développement psychomoteur associé à une microcéphalie.

## Conclusion

Le syndrome de West est une encéphalopathie épileptique du nourrisson avec des caractéristiques cliniques et électroencéphalographiques spécifiques et un mauvais pronostic. La reconnaissance précoce de la maladie, une évaluation diagnostique et un traitement approprié peuvent permettre à certains enfants d'atteindre le contrôle des crises et à mener une vie normale ou au moins beaucoup améliorée.
